# A gene signature predicts response to neoadjuvant chemotherapy in triple-negative breast cancer patients

**DOI:** 10.1042/BSR20190414

**Published:** 2019-05-10

**Authors:** Tianzhi Zheng, Zhiyuan Pang, Zhao Zhao

**Affiliations:** Department of Breast Surgery, Cancer Hospital of China Medical University, Liaoning Cancer Hospital and Institute, China

**Keywords:** gene signature, neoadjuvant chemotherapy, ROC curve, triple-negative breast cancer

## Abstract

Triple-negative breast cancer (TNBC) accounts for approximately 15% of all breast cancer cases. TNBC is highly aggressive and associated with poor prognosis. The present study aimed to compare gene expression between TNBC patients with pathological complete response (pCR) and those with not complete response (nCR) to neoadjuvant chemotherapy. Microarray data of 16 TNBC patients received neoadjuvant chemotherapy were identified from the Gene Expression Omnibus database and 10 patients of them had pCR. We found that 250 coding genes and 155 long noncoding RNAs (lncRNAs) were statistically differentially expressed between patients with pCR and nCR. Receiver operator characteristic curve and area under the curve (AUC) were calculated to assess predictive value of differentially expressed genes. A gene signature of three coding genes and two lncRNA was developed: 2.318*TCF3 + 7.349*CREB1 + 0.891*CEP44 + 0.091*NR_023392.1 + 1.424*NR_048561.1 − 106.682. The gene signature was further validated and had an AUC = 0.829. In summary, we profiled gene expression in pCR patients and developed a gene signature, which was effective to predict pCR among TNBC patients received neoadjuvant chemotherapy.

## Background

Breast cancers are quite heterogeneous since they have variable biological types and have different clinical prognoses and therapeutic responses [1]. Triple negative breast cancer (TNBC) refers to breast cancer that lacking estrogen receptors (ER), progesterone receptors (PR), and HER2 (ERBB2) expression. TNBC accounts for approximately 15% of total invasive breast cancers, which has a higher rate in young African-American women, and TNBC is in general of a higher grade and most of TNBC patients show a signature of basaloid gene expression [2]. Because of the aggressive feature of TNBC than other breast cancer subtypes, TNBC is correlated with early recurrence as well as more frequent distant blood metastasis; therefore, TNBC patients usually have poor overall prognosis. Lehmann and colleagues have identified six subtypes of TNBC with gene expression profiles [3], and they concluded these subtypes might have distinct phenotypes and variable sensitivity to chemotherapy [4].

Neoadjuvant chemotherapy (NAC) refers to administration of chemotherapeutic drugs before surgical resection aiming to decrease the size of breast cancer mass, allowing the planned surgical procedure [5]. Pathologic complete response (pCR) to NAC is defined as the absence of residual invasive tumor tissue from both breast and axilla after neoadjuvant chemotherapy. Many clinical studies have demonstrated NAC would decrease cancer recurrence rate and show a favorable long-term survival in patients achieving pCR to neoadjuvant treatment compared with those have residual tumor tissues after therapy [6,7]. However, more than half of patients with TNBC do not have pCR and have even worse outcomes. Thus, it is essential to develop effective biomarkers to identify patients who will benefit from NAC.

In the present study, we analyzed microarray data of TNBC patients received neoadjuvant chemotherapy and developed a gene signature to predict response to neoadjuvant chemotherapy.

## Materials and methods

### Identifying eligible dataset with TNBC patients received neoadjuvant chemotherapy

We searched the Gene Expression Omnibus (GEO) database to identify eligible dataset included TNBC patients received NAC. The search was limited to Affymetrix human genome U133 plus2 microarray platform, since this microarray platform is widely used, and this microarray platform includes 54,000 probe sets covering the majority of human genome. The following criteria was used to filter potential datasets: (1) HG-U133 plus2 microarray platform was used, (2) including TNBC patients received NAC, (3) ≥5 patients with pCR or not complete response (nCR), (4) ER, PR, and HER status and response to NAC were available. Finally, we found two eligible datasets: GSE50948 [8] and GSE32646 [9]. The GSE50948 dataset includes 156 patients and GSE32646 dataset consists of 115 patients. The GSE50948 dataset was used to investigate differentially expressed genes between pCR and nCR.

### Analysis of microarray data

We used the online GEO2R tool to calculate differentially expressed genes (http://www.ncbi.nlm.nih.gov/geo/geo2r/). To achieve long noncoding RNA (lncRNA) expression in TNBC patients, we download annotation file of HG-U133 Plus 2.0 probe set from BioMart data portal (http://asia.ensembl.org/biomart/martview/). Each probe is correlated with a probe ID, transcript ID, gene symbol, and other information. We download probe ID of Affymatix microarray as well as RefSeq transcript ID and probes with RefSeq transcript ID begin with “NR_” and “XR_” was annotated as lncRNA.

### Bioinformatic analyses

The Database for Annotation, Visualization, and Integrated Discovery (DAVID) website (https://david.ncifcrf.gov/home.jsp) was used to perform function enrichment analyses of Gene Ontology (GO) and pathways for coding genes. Differentially expressed lncRNAs were clustered with one minus correlation and average linkage methods by the Cluster 3.0 software.

### Statistical analyses

We compared continuous variables using Student’s *t*-test and a two-tailed *P* value <0.05 was considered as statistically significant. Receiver operating characteristic curves were constructed to assess sensitivity and specificity of the gene signature, and respective area under the curve (AUC) with 95% confidential interval (CI) were also calculated. Statistical analyses were conducted with SPSS software (version 18.0; SPSS Institute Inc., Chicago, IL, U.S.A.).

## Results

### Baseline information of TNBC patients.

Microarray data of 16 TNBC patients were retrieved and analyzed. The 16 patients aged from 30 to 69 years, among them 5 had pCR and 11 have nCR to neoadjuvant chemotherapy. The neoadjuvant chemotherapy regimen was doxorubicin/paclitaxel followed by cyclophosphamide/methotrexate/fluorouracil.

### Differentially expressed genes in pCR patients

**Figure 1:**
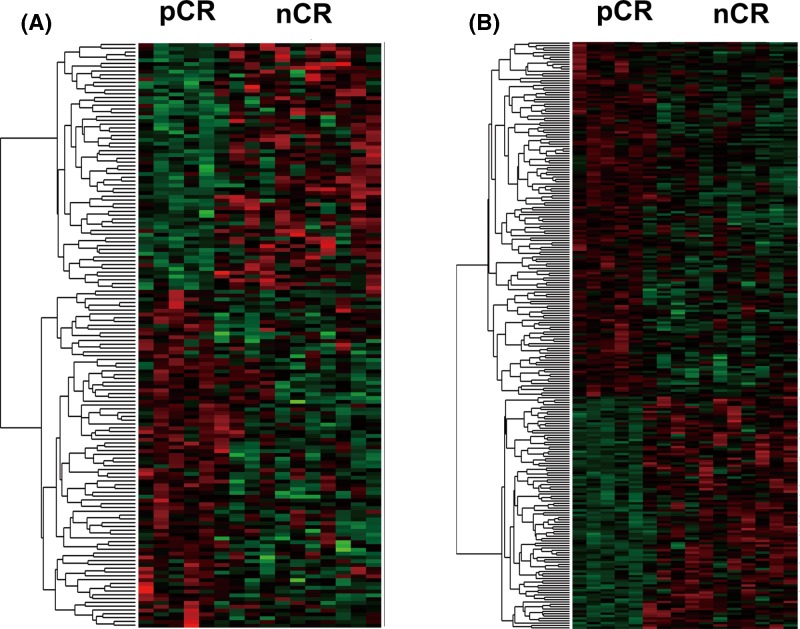
Differentially expressed lncRNAs (**A**) and coding genes (**B**) between pCR and nCR patients. Red: up-regulated genes in pCR; green: down-regulated genes in pCR

We first compared differentially expressed coding genes and long noncoding RNAs between TNBC patients with pCR and nCR to neoadjuvant chemotherapy. After annotation of microarray probes, we found 155 differentially expressed lncRNAs in pCR patients, including 90 up-regulated and 65 down-regulated lncRNAs. A total of 151 coding genes were up-regulated in pCR and 99 were down-regulated in patients with pCR compared with those with nCR (Figure 1). Differentially expressed and lncRNAs were provided in Supplementary Table S1 and differentially expressed coding genes were shown in Supplementary Table S2.

**Figure 2:**
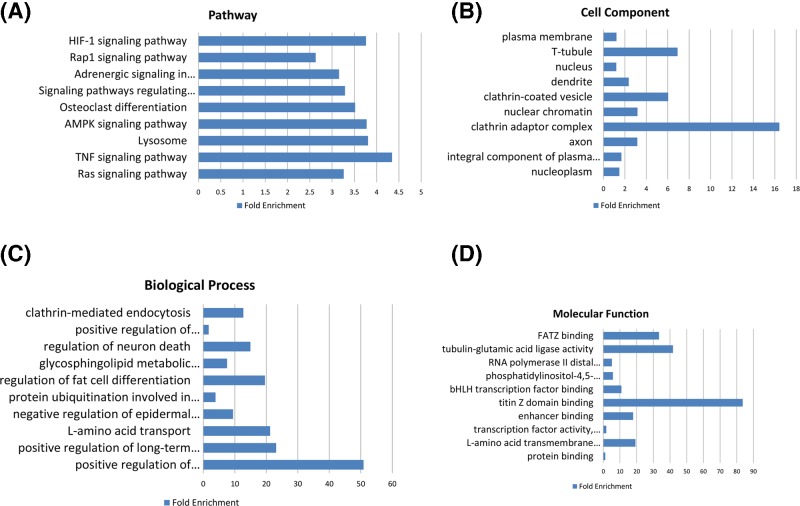
Function enrichment analysis of differentially expressed coding genes. Pathway enrichment analyses (**A**); Gene Ontology analyses of cell component (**B**), biological process (**C**), and molecular function (**D**)

Potential molecular function of these differentially expressed coding genes was further analyzed (Figure 2). Functional enrichment analyses suggested that the differentially expressed genes were involved in Ras signaling pathway, TNF signaling pathway, and lysosome. Positive regulation of hematopoietic stem cell proliferation, positive regulation of long-term synaptic potentiation, and L-amino acid transport were the most enriched biological processes. Clathrin adaptor complex, T-tubule, and clathrin-coated vesicle were the most enriched cell components. Titin Z domain binding, tubulin-glutamic acid ligase activity, and FATZ binding were the most enriched molecular functions.

**Figure 3:**
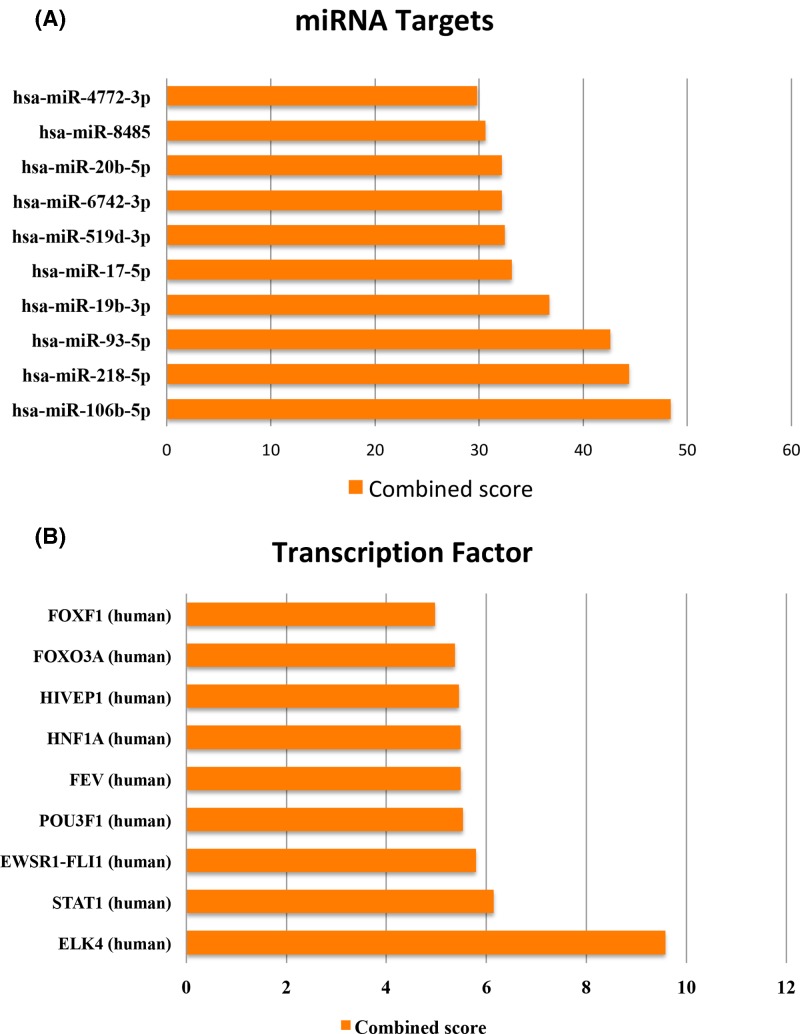
Function enrichment analysis of differentially expressed coding genes. Enrichment of miRNA target sites (**A**) and targets of transcription factors (**B**)

We also analyzed the potential transcription regulation of these differentially expressed genes according to the online tool, Enrichr [10,11]. Target sites of microRNA (miRNA) and transcription factors were analyzed. As shown, the most enriched were target sites of miR-106b-5p, miR-218-5p, miR-93-5p, miR-19b-3p, miR-17-5p, miR-519d-3p, miR-6742-3p, miR-20b-5p, miR-8485, and miR-4772-3p (Figure 3A). For target sites of transcription factors, the most enriched were ELK4, STAT1, EWSR1-FLI1, POU3F1, FEV, HNF1A, HIVEP1, FOXO3A, and FOXF1 (Figure 3B).

### A gene signature predicts response to NAC

**Figure 4:**
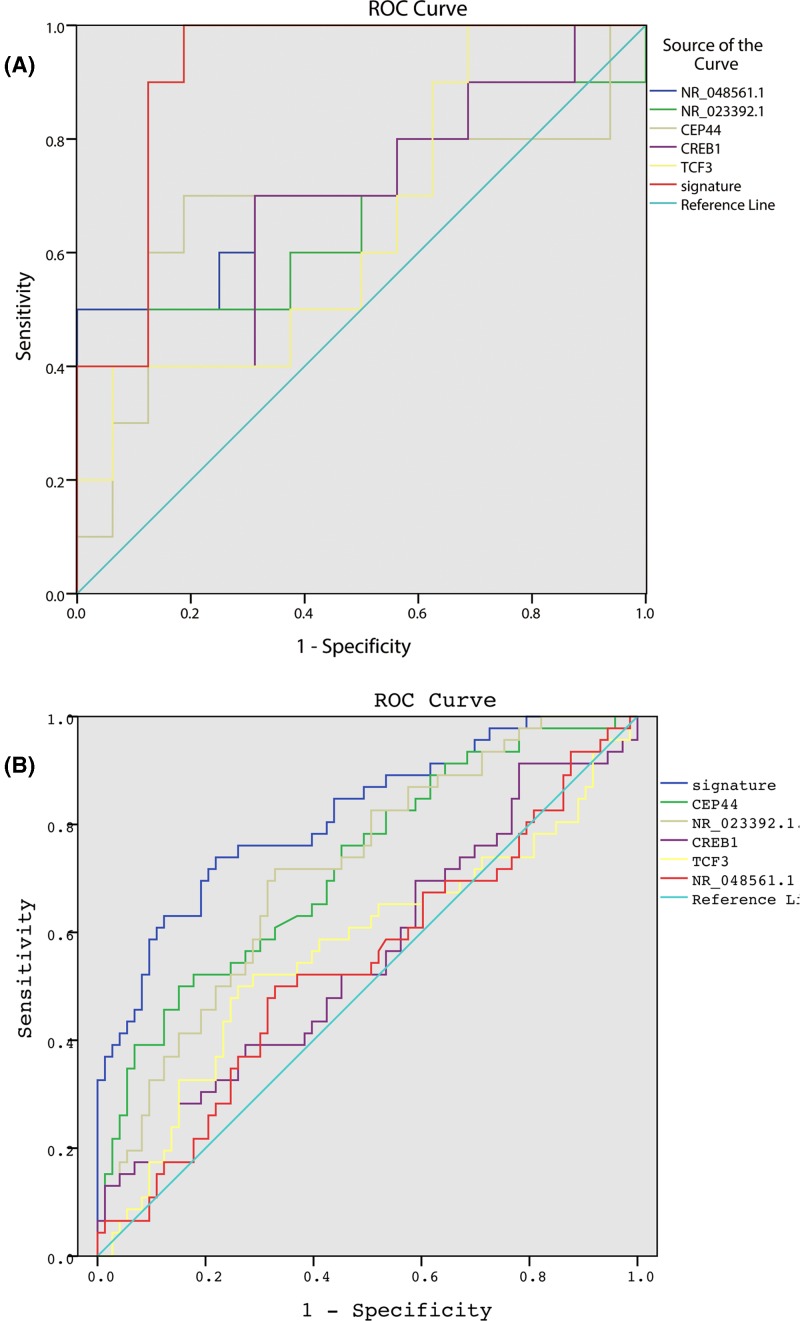
Receiver operative curve of the gene signature (TCF3, CREB1, CEP44, NR_023392.1, and NR_048561.1) in GSE32646 dataset (**A**) and GSE109677 dataset (**B**)

To identify potential biomarkers to predict response to neoadjuvant chemotherapy, we first selected the top 20 differentially expressed coding genes and lncRNAs, respectively, and receiver operation curve was performed for each gene. Intriguingly, most genes showed excellent predictive efficiency with AUC of 1, which may be caused by that the sample size was too small. Then, we further investigated the predictive values of 40 genes in the GSE32646 microarray cohort, and 2 coding genes and 3 lncRNAs showed good predictive efficacy. Thus, we developed a gene signature of 2 coding genes and 3 lncRNAs: 2.318*TCF3 + 7.349*CREB1 + 0.891*CEP44 + 0.091*NR_023392.1 + 1.424*NR_048561.1 − 106.682. As shown in Figure 4A, the gene signature had effective predictive capacity with AUC of 0.919 in the GSE32646 dataset. The sample size of GSE50948 and GSE32646 was not enough for validation, thus, we found an independent cohort (the GSE106977 cohort [12]) to validate this gene signature, which was based on Affymetrix Human Transcriptome Array 2.0 platform and had 117 patients. The good predictive efficacy was also validated in the GSE106977 dataset with AUC = 0.829 (Figure 4B).

## Discussion

In the present study, we found a gene signature of 2 coding genes and 3 lncRNAs could predict pCR to neoadjuvant chemotherapy in patients with TNBC.

Noncoding RNAs were recently found to be important players in cancer progression, metastasis, and chemotherapy resistance [13–15]. Of these, long noncoding RNAs are believed to play major regulatory roles and could be sensitive biomarkers for survival [16–18]. HOTAIR is a well-known lncRNA that was first characterized in breast cancer [19]. Various reports have demonstrated that lncRNAs could be effective biomarkers in breast cancer. For TNBC, few lncRNAs have been used to predict pCR to neoadjuvant chemotherapy. In the present study, we identified 155 lncRNAs differentially expressed between pCR and nCR TNBC patients and developed a gene signature consists of 2 lncRNAs and 3 coding genes. This gene signature showed good performance.

Neoadjuvant chemotherapy has become more common for patients with operable disease, especially in patients with TNBC [4,20,21], while it was initially used only for locally advanced or inflammatory breast cancer. TNBC is an aggressive subtype of breast cancer with a heterogeneous response to therapy [4,20]. Since pCR occurs in only 40–60% of TNBC patients who received neoadjuvant chemotherapy, it is urgent to develop effective biomarkers specific for TNBC patients. Many efforts have been made to identify effective biomarkers. Ki-67 expression was reported associated with response to neoadjuvant chemotherapy [22]. García-Vazquez R found 4 miRNAs (miR-30a, miR-9-3p, miR-770, and miR-143-5p) were associated with pCR to neoadjuvant chemotherapy in TNBC patients [23], while they did not test the predictive efficacy of the 4 miRNAs as a gene signature. Jiang Yizhou also conducted microarray analyses of TNBC patients and identified an integrated mRNA-lncRNA signature of 3 coding genes and 2 lncRNAs (CHRDL1, FCGR1A, RSAD2, HIF1A-AS2, AK124454) [24]. The AUC of Jiang’s signature to predict pCR after neoadjuvant chemotherapy was 0.661, quite lower than our gene signature (0.661 vs. 0.829). However, Jiang’s microarray data (GSE76250) did not provide enough clinical data, such as response to neoadjuvant chemotherapy, on the GEO website [24]; we were unable to validate our gene signature in their dataset.

Our gene signature included three coding genes: TCF3, CREB1, and CEP44. TCF3 is a member of the Wnt pathway-associated TCF/LEF transcription factor family [25]. TCF3 plays important roles in embryonic development, and regulates the identity and function of epidermal and embryonic stem cells. Evidence has demonstrated that TCF3 is recurrently up-regulated in cancers and promotes proliferation and metastasis [26]. CREB1 belongs to the basic leucine zipper (bZIP) family, which is a well-characterized transcription factor that mediates the transduction between the upstream signal and downstream gene transcription [27]. Aberrant expression of CREB1 has been observed in various kinds of cancers, including breast cancer [28]; and CREB1 is also involved in tumor proliferation, invasion, and metastasis [29]. CEP44 is a centrosomal protein while its role in cancers is still unclear. As for the two lncRNA transcripts, NR_023392.1 and NR_048561.1, no reports have been found.

To summary, in the present study, we compared coding and lncRNA expression in TNBC patients received neoadjuvant chemotherapy. An integrated gene signature of three coding genes (TCF3, CREB1, and CEP44) and two lncRNAs (NR_023392.1 and NR_048561.1) could effectively predict pCR to neoadjuvant chemotherapy.

## Supporting information

**Supplementary Table S1:** Differentially expressed lncRNAs between pCR and nCR

**Supplementary Table S2:** Differentially expressed coding genes between pCR and nCR

## Abbreviations

AUC: area under the curve
lncRNA: long noncoding RNA
NAC: neoadjuvant chemotherapy
nCR: not complete response
pCR: pathological complete response
TNBC: triple-negative breast cancer
