# Supplementary material for: A gene signature predicts response to neoadjuvant chemotherapy in triple-negative breast cancer patients
Source: Biosci Rep. 2019 May 10;39(5):BSR20190414. doi: 10.1042/BSR20190414 (PMC6509059; doi:10.1042/BSR20190414)
Supplement: Supplementary file 1 [file bsr20190414_Supp1.pdf]

Supplementary Table 1. Differentially expressed lncRNAs between pCR and nCR

Transcript ID	P.Value	logFC
NR_037195.1	0.001646	0.66594521
NR_145481.1	0.002008	0.3771513
NR_048561.1	0.002099	0.47063902
NR_103480.1	0.002478	-0.96602788
NR_130132.1	0.002746	-0.59755152
NR_037914.1	0.002781	0.80504051
NR_038895.1	0.003172	0.40920889
NR_037713.1	0.003317	-1.38981506
NR_073461.1	0.00424	0.73797038
NR_103508.1	0.004628	-0.72553205
NR_158174.1	0.005287	0.36094143
NR_023392.1	0.005303	-1.21335233
NR_027250.2	0.006698	0.37605004
NR_121659.1	0.007008	0.48134803
NR_103726.1	0.007309	-0.61429391
NR_039982.1	0.007608	0.34748741
NR_130754.1	0.008067	0.38550038
NR_109949.1	0.008194	0.42246433
NR_103869.1	0.008406	0.38574871
NR_028099.1	0.008979	0.39858454
NR_026598.1	0.009234	-0.61845043
NR_125333.1	0.009239	-0.49917742
NR_049780.1	0.009467	0.35797878
NR_152815.1	0.009562	-0.81672368
NR_033684.1	0.009791	-0.55448721
NR_104448.1	0.010156	0.80269984
NR_130136.1	0.011524	-0.6791002
NR_046310.1	0.011985	-0.57519073
NR_110549.1	0.012881	0.41254028
NR_028024.1	0.013036	-0.6002591
NR_110319.1	0.013951	0.96596364
NR_047685.1	0.014024	0.33573129
NR_110105.1	0.0148	-0.49758783
NR_111980.1	0.014878	-0.97023434
NR_016021.1	0.015207	0.40050022
NR_046172.1	0.015861	-0.80518747
NR_125897.1	0.016152	0.37449148
NR_045090.1	0.016809	-0.59470172
NR_003272.2	0.016915	-0.72467339

NR_051952.1	0.01709	-0.75193519
NR_037687.1	0.017414	-0.98706298
NR_104103.1	0.017429	-0.44445427
NR_045763.1	0.017493	0.33457885
NR_104233.1	0.017877	0.64852787
NR_126093.1	0.018178	0.33881687
NR_073070.1	0.018496	-1.40308568
NR_003144.1	0.01868	0.48803401
NR_026752.1	0.018842	-1.09710017
NR_130967.1	0.019024	-0.71930871
NR_037942.1	0.019043	-0.73220245
NR_103469.1	0.019068	0.49244032
NR_122106.1	0.019196	0.84785951
NR_102422.1	0.019532	0.89956661
NR_038375.1	0.019739	0.31308247
NR_026828.1	0.019832	0.45625813
NR_052023.1	0.019837	0.3936943
NR_027668.1	0.020088	-1.20929699
NR_033404.1	0.020118	0.33074956
NR_036542.1	0.020945	0.42991695
NR_002947.2	0.021492	0.35924236
NR_028343.1	0.02164	-0.60454621
NR_103526.1	0.021651	0.31804748
NR_046331.1	0.022059	0.44904262
NR_073120.1	0.022097	-0.96092728
NR_027654.1	0.022129	0.78736399
NR_109978.1	0.022231	0.41378408
NR_037942.1	0.022303	-0.65535629
NR_003089.1	0.022558	0.43344103
NR_046395.1	0.022595	0.58731679
NR_028085.1	0.023129	0.53590812
NR_110242.1	0.023138	0.54481266
NR_126500.1	0.02374	0.31789754
NR_030725.1	0.024465	0.31818415
NR_073018.1	0.024567	0.32987689
NR_038135.2	0.02501	0.34687978
NR_026778.1	0.025162	-0.87285724
NR_111917.1	0.025283	0.42507015
NR_030737.1	0.025404	-0.93681417
NR_036541.1	0.025676	-0.51350824
NR_040016.1	0.02626	-0.83080858
NR_036510.1	0.026365	0.79175104
NR_028406.1	0.026542	-0.58927235

NR_102347.1	0.027157	-0.89531198
NR_126036.1	0.02784	-0.33269119
NR_027384.1	0.028337	0.27601134
NR_028035.2	0.029161	-0.85032547
NR_033302.1	0.029245	-0.74135742
NR_030761.1	0.029347	-0.601602
NR_036505.1	0.029981	0.32147129
NR_015434.1	0.030073	0.3389628
NR_027142.1	0.030371	0.45802125
NR_027032.1	0.031328	0.41441263
NR_047516.1	0.03161	0.59636625
NR_037162.1	0.031935	0.35327812
NR_104244.1	0.032882	0.58857738
NR_037918.2	0.032902	-0.64426642
NR_029423.1	0.033443	-1.06385239
NR_104331.1	0.034275	0.74721934
NR_002724.2	0.034664	0.27761433
NR_036569.1	0.034694	0.50066733
NR_130317.1	0.035056	0.35703807
NR_002798.1	0.035103	0.26892906
NR_104463.1	0.035324	0.26415702
NR_104122.1	0.035327	0.35088687
NR_037591.1	0.035406	-1.14577291
NR_023351.2	0.036156	0.42626329
NR_046335.1	0.036185	-0.30033574
NR_037667.1	0.03636	-0.64260315
NR_122031.1	0.03661	0.29335495
NR_045818.1	0.036707	-1.22807388
NR_045672.1	0.037087	-0.35494805
NR_002796.2	0.03731	0.31493392
NR_130728.1	0.038051	0.28017219
NR_073155.1	0.038081	-0.78017025
NR_125360.1	0.038568	0.32881355
NR_125337.1	0.038833	-0.41879069
NR_103826.1	0.039293	-0.50286697
NR_104020.1	0.039318	-0.69470594
NR_046333.1	0.03943	0.32596818
NR_047690.1	0.039461	0.47174069
NR_104292.1	0.039497	0.38167533
NR_126502.1	0.039637	0.44360183
NR_046331.1	0.040134	0.34435854
NR_104200.1	0.040759	0.34790401
NR_047582.1	0.040797	-0.44619399

NR_110332.1	0.040942	-0.59680116
NR_033464.1	0.042353	0.57406821
NR_028057.1	0.04241	0.28153949
NR_030765.1	0.042492	0.29703078
NR_037791.1	0.042645	0.30535607
NR_026665.1	0.04269	-0.72290565
NR_104032.1	0.042845	1.02752725
NR_036633.1	0.043129	-0.61462516
NR_036641.1	0.043165	0.52504602
NR_037942.1	0.043437	-0.59452487
NR_073108.1	0.043438	0.7697725
NR_046417.1	0.044123	0.43273914
NR_030724.1	0.044277	-0.41451022
NR_027962.1	0.044457	-0.99374101
NR_023349.2	0.044777	-0.56340308
NR_038271.1	0.045948	-0.36906346
NR_003505.3	0.046258	0.33270224
NR_120681.1	0.047044	-0.69049628
NR_026963.1	0.047379	0.24971244
NR_073546.1	0.047517	0.32090289
NR_027840.1	0.047612	-0.66301799
NR_003267.1	0.047971	0.25848361
NR_109991.1	0.048288	0.2813425
NR_003109.1	0.048772	-0.42800143
NR_103464.1	0.048903	-0.5553307
NR_027397.1	0.048924	-0.59765669
NR_046672.1	0.048983	0.34085956
NR_027767.1	0.049295	0.60114625
NR_073366.1	0.049529	-0.57859708
NR_024047.1	0.049871	0.31932883

Supplementary Table 2. Differentially expressed coding genes between pCR and nCR

Gene symbol	P.Value	logFC	Gene.title
MPZL3	0.000102	0.67621743	myelin protein zero like 3
NSFL1C	0.000161	0.80677465	NSFL1 cofactor
PTPN2	0.000169	-0.96517728	protein tyrosine phosphatase, non-receptor type 2
TTLL7	0.000279	1.05016167	tubulin tyrosine ligase like 7
CREB1	0.000293	-1.0169655	cAMP responsive element binding protein 1
FANCF	0.000301	-0.95034838	Fanconi anemia complementation group F
CEP44	0.000306	0.64175134	centrosomal protein 44
SAYSD1	0.000328	0.8428069	SAYSVFN motif domain containing 1
LOC389765	0.000341	-1.33223042	kinesin family member 27 pseudogene
USP34	0.00038	-1.23911822	ubiquitin specific peptidase 34
GLS	0.00038	-0.96630802	glutaminase
SLC2A4	0.000413	0.5377713	solute carrier family 2 member 4
LOC105371352	0.00043	0.478354	uncharacterized LOC105371352
PGGT1B	0.000762	1.11999862	protein geranylgeranyltransferase type I subunit beta
HNRNPU	0.000894	-1.23336074	heterogeneous nuclear ribonucleoprotein U
SLC12A9	0.000922	-1.26601107	solute carrier family 12 member 9
GS1-124K5.4	0.001185	0.63125241	uncharacterized LOC100289098
TCF3	0.001194	-1.01317597	transcription factor 3
RALB	0.001254	0.61976104	RALB Ras like proto-oncogene B
CYB561	0.001371	0.57293509	cytochrome b561
MAGI1	0.00138	0.5556342	membrane associated guanylate kinase, WW and PDZ domain containing 1
CDRT1	0.001445	0.58246033	CMT1A duplicated region transcript 1
SLC7A8	0.001448	0.73009006	solute carrier family 7 member 8
ANKRD12	0.001462	-2.06275873	ankyrin repeat domain 12
SLC39A10	0.001547	-1.15992914	solute carrier family 39 member 10
FOXN3	0.001561	0.68903409	forkhead box N3
SNORD10	0.001575	-0.64971186	small nucleolar RNA, C/D box 10
LINC00607	0.001646	0.66594521	long intergenic non-protein coding RNA 607
DRAIC	0.001673	1.25818517	downregulated RNA in cancer, inhibitor of cell invasion and migration
KCNJ2	0.001722	-1.22291121	potassium voltage-gated channel subfamily J member 2
EXD3	0.001728	0.44227907	exonuclease 3'-5' domain containing 3
KDM5A	0.001741	0.89087181	lysine demethylase 5A
PPIAL4C	0.001742	0.54252167	peptidylprolyl isomerase A like 4C
NMNAT1	0.001765	-0.58962268	nicotinamide nucleotide adenylyltransferase 1
AP2A2	0.001792	0.55529614	adaptor related protein complex 2 alpha 2 subunit
LOC100631378	0.001888	0.67251655	uncharacterized 100631378
MUC4	0.001943	1.30765223	mucin 4, cell surface associated
PARP6	0.001965	-0.72996651	poly(ADP-ribose) polymerase family member 6
PSG11	0.002044	0.56196488	pregnancy specific beta-1-glycoprotein 11

LOC285178	0.0021	0.56448064	uncharacterized LOC285178
GPR101	0.002232	0.58524536	G protein-coupled receptor 101
ARID4B	0.002327	-0.6229422	AT-rich interaction domain 4B
SQSTM1	0.00235	0.46078951	sequestosome 1
MARK1	0.002623	1.03309076	microtubule affinity regulating kinase 1
ACVR1B	0.002627	0.56597027	activin A receptor type 1B
GLDN	0.002628	0.45758731	gliomedin
KCNK1	0.002689	-0.9543384	potassium two pore domain channel subfamily K member 1
EPB41L5	0.002768	-0.68800344	erythrocyte membrane protein band 4.1 like 5
TMEM107	0.002858	-1.5773623	transmembrane protein 107
PPP2R5E	0.002884	-0.94135307	protein phosphatase 2 regulatory subunit B'epsilon
PHC3	0.00292	0.66912703	polyhomeotic homolog 3
CCDC154	0.002933	0.57484187	coiled-coil domain containing 154
SLC38A1	0.00297	0.60298275	solute carrier family 38 member 1
ZNF780A	0.003014	-2.00588291	zinc finger protein 780A
FBXO42	0.003051	0.54830476	F-box protein 42
SMARCB1	0.003076	-0.95791751	SWI/SNF related, matrix associated, actin dependent regulator of chromatin, subfamily b, member 1
HRH1	0.003089	0.6575804	histamine receptor H1
EXPH5	0.003157	0.61700322	exophilin 5
ACTR3C	0.003179	0.58554111	ARP3 actin-related protein 3 homolog C
SLC2A12	0.003243	0.60631795	solute carrier family 2 member 12
RAP1GDS1	0.003244	-0.95358695	Rap1 GTPase-GDP dissociation stimulator 1
CASP7	0.003304	-0.62313428	caspase 7
GPR75	0.003306	-0.79582434	G protein-coupled receptor 75
PIK3R1	0.003338	-1.49761877	phosphoinositide-3-kinase regulatory subunit 1
LOC283737	0.003418	0.5898756	uncharacterized LOC283737
CNST	0.003424	-0.65764262	consortin, connexin sorting protein
SFXN1	0.003454	-0.64066823	sideroflexin 1
AKT3	0.003528	-0.4952383	AKT serine/threonine kinase 3
SNORA21	0.003545	0.96168848	small nucleolar RNA, H/ACA box 21
DSPP	0.003549	0.63425177	dentin sialophosphoprotein
MBNL3	0.00355	0.55945048	muscleblind like splicing regulator 3
ITCH	0.003555	0.80037154	itchy E3 ubiquitin protein ligase
SDPR	0.003565	0.5261997	serum deprivation response
FLT1	0.003577	1.05704093	fms related tyrosine kinase 1
N4BP2L2	0.003644	-1.18609692	NEDD4 binding protein 2 like 2
FOXJ1	0.003648	0.41675994	forkhead box J1
COMMD10	0.003693	0.96129514	COMM domain containing 10
GGA2	0.003735	-0.51083678	golgi associated, gamma adaptin ear containing, ARF binding protein 2
SLC25A11	0.003764	1.48865305	solute carrier family 25 member 11
TATDN3	0.003765	-0.74567903	TatD DNase domain containing 3
ATP2A3	0.00386	0.86411966	ATPase sarcoplasmic/endoplasmic reticulum Ca2+ transporting 3

CYP19A1	0.003971	0.47958921	cytochrome P450 family 19 subfamily A member 1
TGIF2LY	0.003996	0.54346061	TGFB induced factor homeobox 2 like, Y-linked
GPR161	0.00405	-1.02000809	G protein-coupled receptor 161
USP4	0.004117	-0.72546575	ubiquitin specific peptidase 4
TCAP	0.004137	0.43613776	titin-cap
SLC44A1	0.004152	-0.83164799	solute carrier family 44 member 1
EIF4EBP2	0.004194	0.92015341	eukaryotic translation initiation factor 4E binding protein 2
TMEM248	0.004262	-0.6153004	transmembrane protein 248
GMPPA	0.0043	-0.41878904	GDP-mannose pyrophosphorylase A
MME	0.004317	0.56741649	membrane metallo-endopeptidase
CEACAM21	0.004371	0.41040542	carcinoembryonic antigen related cell adhesion molecule 21
NAT2	0.004402	0.47994117	N-acetyltransferase 2
PCNX3	0.004445	0.5972109	pecanex homolog 3 (Drosophila)
GJD3	0.004463	0.51886215	gap junction protein delta 3
RFX2	0.004515	0.46407575	regulatory factor X2
TNS4	0.00459	0.57035243	tensin 4
NADK	0.004611	-0.67146245	NAD kinase
FOXH1	0.004652	-1.1414079	forkhead box H1
ARSK	0.004657	0.48165728	arylsulfatase family member K
TRIM25	0.004662	0.47687143	tripartite motif containing 25
RNF126	0.004695	0.42382251	ring finger protein 126
HNRNPCL4	0.004782	0.36135699	heterogeneous nuclear ribonucleoprotein C-like 4
SLC16A5	0.004802	0.56630724	solute carrier family 16 member 5
GDE1	0.00481	-0.87342905	glycerophosphodiester phosphodiesterase 1
SLC19A3	0.004814	0.62420624	solute carrier family 19 member 3
DNAJB13	0.004825	0.49077523	DnaJ heat shock protein family (Hsp40) member B13
DICER1	0.004866	0.56644252	dicer 1, ribonuclease III
C7orf43	0.004947	-0.42041484	chromosome 7 open reading frame 43
TRIM16L	0.004962	0.99839951	uncharacterized LOC101930303
CDC20B	0.005086	1.17196393	cell division cycle 20B
SDK2	0.005116	0.46408752	sidekick cell adhesion molecule 2
CNPY3	0.005125	-0.60314706	canopy FGF signaling regulator 3
LOC101928730	0.00514	0.45282751	uncharacterized LOC101928730
FAM178B	0.00522	0.34314013	family with sequence similarity 178 member B
PARD3B	0.005292	0.53106292	par-3 family cell polarity regulator beta
TMPRSS12	0.005333	1.07855264	transmembrane protease, serine 12
CDYL2	0.00535	0.97091013	chromodomain Y-like 2
BACH2	0.005353	0.68887252	BTB domain and CNC homolog 2
RBM4	0.005381	-0.83141664	RBM14-RBM4 readthrough/
PDCD2	0.0054	0.62236352	programmed cell death 2
TANK	0.005441	-0.97792417	TRAF family member associated NFKB activator
IGH	0.005457	0.74647365	immunoglobulin heavy locus
HIST1H3F	0.005462	-0.87271991	histone cluster 1, H3f

LOC101929872	0.005463	0.54129319	uncharacterized LOC101929872
FCHO1	0.005523	0.37452593	FCH domain only 1
LILRA2	0.005553	0.79075299	leukocyte immunoglobulin like receptor A2
MYCBP	0.005578	0.99452232	GJA9-MYCBP readthrough
RAB28	0.005598	-0.47040542	RAB28, member RAS oncogene family
RASAL1	0.005642	0.46951657	RAS protein activator like 1
THRA	0.00568	0.43863612	thyroid hormone receptor, alpha
ALMS1	0.005711	-0.96666562	ALMS1, centrosome and basal body associated protein
FTSJ1	0.005774	0.60171195	FtsJ RNA methyltransferase homolog 1 (E. coli)
HOXB5	0.005815	0.51606912	homeobox B5
CCDC181	0.005891	0.46741429	coiled-coil domain containing 181
RUNX2	0.005974	1.51743317	runt related transcription factor 2
COX15	0.00598	0.52252235	COX15, cytochrome c oxidase assembly homolog
CNOT7	0.006025	-0.556832	CCR4-NOT transcription complex subunit 7
SATB1	0.006044	0.64876156	SATB homeobox 1
KIAA1211L	0.006045	0.4258144	KIAA1211 like
ZNF282	0.006055	0.4828297	zinc finger protein 282
HIP1	0.00607	0.59031717	huntingtin interacting protein 1
JKAMP	0.006089	-0.8458754	JNK1/MAPK8-associated membrane protein
CACNB2	0.006103	-1.17121657	calcium voltage-gated channel auxiliary subunit beta 2
LOC153577	0.006125	0.44786223	uncharacterized LOC153577
KIRREL	0.006166	-1.37329508	kin of IRRE like (Drosophila)
PRRC2C	0.006202	-0.67624385	proline rich coiled-coil 2C
CDH24	0.006222	0.84979927	cadherin 24
SMC2	0.006356	0.75363945	structural maintenance of chromosomes 2
SPOP	0.00637	0.4330572	speckle type BTB/POZ protein
OSBP2	0.006385	0.38343846	oxysterol binding protein 2
APOE	0.006404	0.61946475	apolipoprotein E
SS18	0.006415	-0.82872114	SS18, nBAF chromatin remodeling complex subunit
CD1B	0.006419	0.52748822	CD1b molecule
CCDC117	0.006509	-0.85703975	coiled-coil domain containing 117
MRPS11	0.00652	0.60593581	mitochondrial ribosomal protein S11
ANK3	0.006554	0.81205404	ankyrin 3, node of Ranvier (ankyrin G)
ZNF621	0.006594	-0.9358599	zinc finger protein 621
STS	0.006631	0.52980355	steroid sulfatase (microsomal), isozyme S
NBN	0.006632	-0.92168976	nibrin
METTL23	0.006702	-0.80019922	methyltransferase like 23
TIA1	0.006725	-0.78453399	TIA1 cytotoxic granule-associated RNA binding protein
IL31RA	0.006765	0.39085712	interleukin 31 receptor A
WDR86	0.006796	0.63000788	WD repeat domain 86
ABL2	0.006824	0.48839208	ABL proto-oncogene 2, non-receptor tyrosine kinase
DUSP18	0.006831	0.51802291	dual specificity phosphatase 18
TARBP1	0.006835	-0.87983277	TAR (HIV-1) RNA binding protein 1

OLFM2	0.006875	0.87301125	olfactomedin 2
PPID	0.006882	-0.63927924	peptidylprolyl isomerase D
MON2	0.006969	-0.97150867	MON2 homolog, regulator of endosome-to-Golgi trafficking
KITLG	0.00698	0.52950798	KIT ligand
CCDC97	0.006989	0.43863584	coiled-coil domain containing 97
SLC35B4	0.007005	0.48957451	solute carrier family 35 member B4
ZNF37BP	0.007007	-1.12723671	zinc finger protein 37B, pseudogene
CMKLR1	0.007133	0.30949928	chemerin chemokine-like receptor 1
NCOA7	0.007147	-0.58680766	nuclear receptor coactivator 7
THEM4	0.007161	-0.78366598	thioesterase superfamily member 4
LOC105374366	0.007176	0.53722123	uncharacterized LOC105374366
LMLN	0.007234	0.57104923	leishmanolysin like peptidase
UBE3B	0.007249	0.49538533	ubiquitin protein ligase E3B
PNPLA3	0.007294	0.46721752	patatin like phospholipase domain containing 3
PLB1	0.0073	0.50173325	phospholipase B1
MTDH	0.0074	-1.34435864	metadherin
SON	0.007482	-0.61042855	SON DNA binding protein
ADAM32	0.007495	0.6462346	ADAM metallopeptidase domain 32
RORC	0.007539	0.43788254	RAR related orphan receptor C
ALKBH8	0.007575	-0.51690241	alkB homolog 8, tRNA methyltransferase
GSE1	0.007657	0.71025944	Gse1 coiled-coil protein
ELMO2	0.007756	-0.40722864	engulfment and cell motility 2
LOC285095	0.007759	0.36888923	uncharacterized LOC285095
SPEN	0.007771	-0.82806585	spen family transcriptional repressor
TCF20	0.00781	-0.78870464	transcription factor 20
ANO7	0.007835	1.20850661	anoctamin 7
SNRK	0.007859	0.51991495	SNF related kinase
ZBTB38	0.007953	0.66207558	zinc finger and BTB domain containing 38
CERK	0.007976	-0.48550195	ceramide kinase
SLC30A5	0.008074	-0.67169739	solute carrier family 30 member 5
PWAR5	0.008083	-0.95621519	Prader Willi/Angelman region RNA 5
PATL1	0.008095	-0.60294863	PAT1 homolog 1, processing body mRNA decay factor
CEACAM6	0.008112	1.96607951	carcinoembryonic antigen related cell adhesion molecule 6
MAFK	0.008146	0.51912763	MAF bZIP transcription factor K
CCNG2	0.008158	0.6221167	cyclin G2
GON4L	0.00818	-0.88975378	gon-4 like
TIAF1	0.008227	-0.56950185	TGFB1-induced anti-apoptotic factor 1
DKFZp564H213	0.008244	-0.55143431	uncharacterized LOC440432
SDHAF4	0.008285	0.72372921	succinate dehydrogenase complex assembly factor 4
PTP4A3	0.008324	-1.32105187	protein tyrosine phosphatase type IVA, member 3
DDX11	0.008335	-1.09003919	DEAD/H-box helicase 11
ZNF385A	0.008371	0.61365913	zinc finger protein 385A
LOC730961	0.0084	0.33946497	uncharacterized LOC730961

LCA5L	0.008408	0.47347349	LCA5L, lebercilin like
PPM1K	0.008417	-0.96368551	protein phosphatase, Mg2+/Mn2+ dependent 1K
LUC7L2	0.008443	-0.90287228	C7orf55-LUC7L2 readthrough
MAT2A	0.008495	-0.7337722	methionine adenosyltransferase 2A
C9orf3	0.008511	-0.43160019	chromosome 9 open reading frame 3
RAB5A	0.008531	0.974627	RAB5A, member RAS oncogene family
RNF115	0.008547	-0.78286567	ring finger protein 115
NAGPA	0.008606	0.47584603	N-acetylglucosamine-1-phosphodiester alpha-N-acetylglucosaminidase
UNK	0.008636	-0.60379696	unkempt family zinc finger
GRIP1	0.008652	-0.96439627	glutamate receptor interacting protein 1
ARID2	0.008657	-0.70201683	AT-rich interaction domain 2
EIF2AK4	0.008674	-0.98926599	eukaryotic translation initiation factor 2 alpha kinase 4
DCC	0.008785	0.33229198	DCC netrin 1 receptor
MXD4	0.008818	0.53819527	MAX dimerization protein 4
ZC3H14	0.008851	-1.03057581	zinc finger CCCH-type containing 14
AP3M1	0.008862	0.4562984	adaptor related protein complex 3 mu 1 subunit
M6PR	0.008944	-1.09755415	mannose-6-phosphate receptor, cation dependent
LOC101930415	0.009	-0.90555283	uncharacterized LOC101930415
LMX1B	0.009045	0.3686186	LIM homeobox transcription factor 1 beta
MCTP1	0.009117	0.77457338	multiple C2 and transmembrane domain containing 1
MKNK1	0.009124	0.77804391	MAP kinase interacting serine/threonine kinase 1
THUMPD1	0.009194	-0.62111673	THUMP domain containing 1
ZNF641	0.009201	-0.87671588	zinc finger protein 641
RAB3IL1	0.009225	0.47271049	RAB3A interacting protein like 1
TPSG1	0.009248	0.39978085	tryptase gamma 1
ZFHX3	0.009297	0.87783293	zinc finger homeobox 3
GLIPR1	0.009319	0.78873007	GLI pathogenesis related 1
MMD	0.009343	0.44725569	monocyte to macrophage differentiation associated
PSG4	0.009407	0.37978358	pregnancy specific beta-1-glycoprotein 4
MED25	0.009477	-0.7835693	mediator complex subunit 25
TRIP12	0.009528	-0.5751403	thyroid hormone receptor interactor 12
ACTN2	0.009561	0.42981852	actinin alpha 2
NEU1	0.009707	0.50370338	neuraminidase 1 (lysosomal sialidase)
ASXL3	0.009738	0.58816344	additional sex combs like 3, transcriptional regulator
NRGN	0.009825	0.42204144	neurogranin
PGM5	0.009924	0.30349646	phosphoglucomutase 5
DBT	0.009939	-0.53422517	dihydrolipoamide branched chain transacylase E2
PBX2	0.009942	-0.78443439	PBX homeobox 2
GPATCH2	0.009949	-0.78806239	G-patch domain containing 2
TIMM23	0.009962	-0.9433154	translocase of inner mitochondrial membrane 23
